# Cardiovascular risk in Cuban adolescents and young adults with congenital adrenal hyperplasia

**DOI:** 10.1186/s12902-023-01499-9

**Published:** 2023-11-02

**Authors:** Tania M. Espinosa Reyes, Alba Katherine Pesántez Velepucha, Julio Oscar Cabrera Rego, Wendy Valdés Gómez, Emma Domínguez Alonso, Henrik Falhammar

**Affiliations:** 1 Department of Pediatric Endocrinology, National Institute of Endocrinology, Havana, Cuba; 2 University Surgical Clinical Hospital Manuel Fajardo, Havana, Cuba; 3 Department of Molecular Medicine and Surgery, Karolinska Institutet, Stockholm, Sweden; 4 Department of Endocrinology, QB85 Karolinska University Hospital, 171 76 Stockholm, Sweden

**Keywords:** 21-hydroxylase deficiency, Endothelial dysfunction, Carotid intima media thickness, 17-hydroxyprogesterone, Blood pressure, Insulin resistance

## Abstract

**Background:**

Hyperandrogenism and supraphysiologic glucocorticoid replacement may lead to subclinical atherosclerosis in people with congenital adrenal hyperplasia (CAH) and predispose the development of cardiovascular diseases from an early age.

**Objectives:**

To determine if cardiometabolic risk factors and subclinical atherosclerosis are more frequent in patients with CAH due to 21-hydroxylase deficiency (21OHD) and if there is an association with clinical, hormonal and treatment of 21OHD.

**Material and methods:**

A descriptive prospective cross-sectional study exploring clinical variables, biochemical, hormonal variables, endothelial dysfunction (flow-mediated dilation < 5%) and carotid intima media thickness (≥ 95 percentile in adolescents and ≥ 75 percentile in adults) and epicardial fat. Adolescents and young patients with 21OHD were compared with controls matched by age, sex, body mass index and Tanner stage.

**Results:**

Forty four subjects (22 with CAH), 36 (82%) females, with a mean age of 17.1 ± 5.5 years (range 10–30 years) were included. Family history revealed diabetes, hypertension, and hypercholesterolemia with high frequencies in both groups. The blood pressure was similar in both groups. Blood glucose levels were lower and triglycerides higher in patient (both *p* < 0.01). Epicardial fat was similar between groups and in patients with CAH it was related to cholesterol levels ​​(*r* = 0.679, *p* < 0.01), time since CAH diagnosis (*r* = 0.462, *p* = 0.03) and glucocorticoid dose (*r* = 0.499, *p* = 0.04). Carotid intima media thickness (CIMT) had a tendency to be increased in patients (*p* = 0.07) and was directly related to 17-hydroxyprogesterone (*r* = 0.510, *p* = 0.018), diastolic blood pressure (*r* = 0.444, *p* = 0.04) and the homeostatic model assessment (HOMA) index (*r* = 0.507, *p* = 0.01). Endothelial dysfunction was not different between groups.

**Conclusions:**

Some cardiometabolic risk factors were increased in patients with CAH and were associated with clinical, hormonal and treatment parameters of CAH. Cardiometabolic risk should be evaluated regularly in patients with CAH.

## Introduction

Congenital adrenal hyperplasia (CAH) refers to a group of inherited enzyme defects in cortisol biosynthesis. It is the most common adrenal disorder in childhood with equal involvement of both sexes and with a very wide spectrum of clinical manifestations [1, 2]. The spectrum of enzyme deficiencies varies from partial to total, with 21-hydroxylase deficiency (21OHD) being the most frequent, accounting for 95–99% of all CAH cases [1–3].

Achieving normal growth and development remains a primary goal of treatment for CAH. Glucocorticoids and, when necessary, mineralocorticoids, are the mainstay of treatment in these patients, both for substitution of deficient steroids and for the purpose of suppressing excessive adrenal androgen production [1, 4].

Patients with CAH are considered to be at higher cardiometabolic risk compared to general population, due to more risk factors such as obesity, insulin resistance hypertension and dyslipidemia. The constellation of these factors, which begins in childhood, could result in endothelial dysfunction and predispose to atherosclerosis, metabolic syndrome and cardiovascular diseases at an early age [5–12].

The exact reasons that explain the increased cardiovascular risk in patients with CAH and the pathophysiology of the metabolic alterations detected are still unknown. The combination of supraphysiological glucocorticoid replacement, hyperandrogenism, and hypofunction of the adrenal medulla due to CAH, an element that has been added in recent years [6, 13, 14], makes these patients more prone to developing cardiovascular risk factors [6, 12].

Atherosclerosis is the most common underlying cause of cardiovascular diseases and is considered the main cause of morbidity and mortality internationally. In addition, it is the main cause responsible for disability and impaired quality of life in all developed countries and in those where infections are not the primarily cause of mortality [7, 10–12].

Subclinical atherosclerosis is defined as the asymptomatic or preclinical phase of atherosclerosis. Markers of subclinical atherosclerosis are a set of anatomical, functional or serological findings that allow diagnosing atherosclerotic disease in the preclinical stage and thus classifying patients into the appropriate risk category [7].

The measurement of the flow-mediated dilation (FMD) of the brachial artery that reflects the endothelial function, as well as the measurement of the carotid intima media thickness (CIMT), are the two most used, simple and non-invasive methods to demonstrate subclinical atherosclerosis and are useful as independent predictors of future cardiovascular events [15]. Endothelial dysfunction and increased CIMT have occasionally been reported in children and young adults with CAH [5, 6].

Epicardial adipose tissue has been identified as an important element in the development of cardiovascular disease and may reflect intra-abdominal visceral fat. Therefore, echocardiographic evaluation of epicardial fat could serve as a reliable marker of visceral adiposity. Epicardial fat is also associated with clinical and anthropometric parameters of the metabolic syndrome; therefore, echocardiographic evaluation of epicardial fat is considered a simple tool to stratify cardiovascular risk in clinical practice [16].

Increased CIMT could be related to chronic gluco- and mineralocorticoid treatment or hyperandrogenism. However, a direct association between these variables in patients with CAH has not been precisely demonstrated, although it has been some indications, especially with blood pressure [8].

It is still not clear whether subclinical atherosclerosis is related to CAH itself, its treatment, or both [1]. Thus, the aims of the current study were to determine if subclinical atherosclerosis was more frequent in adolescents and young adults with 21OHD and its associations with clinical, hormonal and treatment parameters.

## Material and methods

This is a descriptive prospective cross-sectional study carried out at the National Institute of Endocrinology (INEN), Havana, Cuba; from January 2017 to July 2020.

All patients with CAH due to 21OHD who attended or were referred to the INEN during the study period were prospectively considered. The inclusion criteria were a diagnosis of CAH due to 21OHD [17], age 10 to 30 years old to reflect adolescents and young adults, and at least 1 year of follow-up data. The exclusion criteria were presence of known cardiovascular disease, e.g., congenital or acquired heart disease, heart failure; or hypertension not related to CAH, presence of other conditions that increase cardiovascular risk, such as diabetes and, primary dyslipidemias and patients where the chromosomal and social sex differed.

Controls were selected from apparently healthy subjects, matched for age, sex, body mass index (BMI) and Tanner stage from nearby primary health care. For the selection of controls, 40 subjects were examined. To exclude the possibility of CAH in controls, serum 17-hydroxyprogesterone (17OHP) levels were measured.

A detailed personal (pathological history, smoking or not) and family history as well as physical examination were carried out at INEN including weight, height, BMI (calculated as weight in kg/height in m^2^), waist circumference and abdominal adiposity (abdominal circumference/height > 0.5). The current age, assigned sex, pubertal stage according to Tanner were determined through questioning and physical examination and they were taken into account in the interpretation of the procedures [18]. Blood pressure (BP) was measured considered the instructions of the Cuban Guide for the prevention, diagnosis and treatment of hypertension where elevated SBP and/or DBP ≥ 90 percentile or BP ≥ 120/80 mmHg were defined as hypertension [19]. The biochemical variables measured were fasting plasma glucose, categorized as hypoglycemia (≤ 3.0 mmol/L), normal (3.1–5.59 mmol/L), impaired fasting glucose (5.6–6.99 mmol/L) and hyperglycemia (≥ 7 mmol/L); fasting insulin (mIU/ml) and the HOMA-IR was calculated to determine insulin resistance (≥ 75 percentile in adolescents and ≥ 2.6 in adults); total cholesterol (TC) (elevated ≥ 5.2 mmol/L);. triglycerides (TG) (elevated ≥ 1.7 mmol/L); HDL cholesterol (HDL) (low < 1.03 mmol/L); and LDL cholesterol (LDL) (elevated ≥ 3.4 mmol/L) [20]. Dyslipidemia was defined as any elevation of TC, TG and LDL or low HDL. LDL was calculated using the Friedewald formula: LDL (mmol/L) = TC—HDL -TG / 2.2, provided that the TG were below 4.5 mmol/L [20]. The atherogenic index was calculated: total cholesterol /HDL (mg/dL) and it was considered normal < 4 [21]. Also, serum testosterone (nmol/L) (RIA KIT, IZOTOP, Budapest, Hungary) and serum 17OHP measured before morning medications (nmol/L)(RIA-CT, DIASource, Ottignies-Louvain-la-Neuve, Belgium). All bloods were measured in the morning after an overnight fast, using and defining hyperandrogenism according to reference levels provided by the manufacturer.

 After this, the participants were transferred to the imaging service of the Manuel Fajardo Clinical Surgical Hospital to determine the FMD of the brachial artery, the presence of epicardial fat and the CIMT. FMD was measured as the percentage changed in brachial artery diameter from baseline in response to the increased flow. Measurement of CIMT was obtained through carotid echo doppler (Aloka alpha 5 equipment with a high frequency linear transducer (5–13 MHz)). Epicardial fat was demonstrated by transthoracic echocardiography with patients in left lateral decubitus and is the echo-lucent area between the epicardium of the right ventricle and parietal pericardium, which is seen as a thick line above the right ventricular free wall on echocardiography. Both measurements (CIMT and epicardial fat) were performed three times. There is yet no definite value considered normal for epicardial adipose tissue (EAT) thickness. There are inconsistencies in the literature regarding EAT thickness. In the present study increased EAT thickness was considered 5 mm or more measured in end-diastole in three cardiac cycles using a parasternal long axis or parasternal short axis view, a threshold for subclinical atherosclerosis as Bertaso et al. suggested in a systematic review [22]. All procedures were performed by a trained cardiologist. The diagnosis of subclinical atherosclerosis was defined as an increased CIMT (≥ 95 percentile in adolescents and ≥ 75 percentile in adults) [23, 24] and/or endothelial dysfunction (FMD < 5%) [25]. All methods were carried out in accordance with relevant guidelines and regulations.

Variables related to CAH was noted such as time since diagnosis and type of glucocorticoid (hydrocortisone and cortisone acetate), which were converted to hydrocortisone equivalences (cortisone acetate 25 mg = hydrocortisone 20 mg) and daily dose of the hydrocortisone equivalents (mg/m^2^).

### Statistical analysis

The mean ± SD or median and range were used and comparing groups was done with the Student's *t* test (or ANOVA) and Mann–Whitney *U* test (or Kruskal–Wallis test) as appropriate. Categorial parameters were compared using Fisher's exact test or Chi-square test, as appropriate. The association of quantitative variables were done ​​using the Pearson or Spearman correlation coefficient, as appropriate. A *p*-value < 0.05 was considered significant.

## Results

**Fig. 1:**
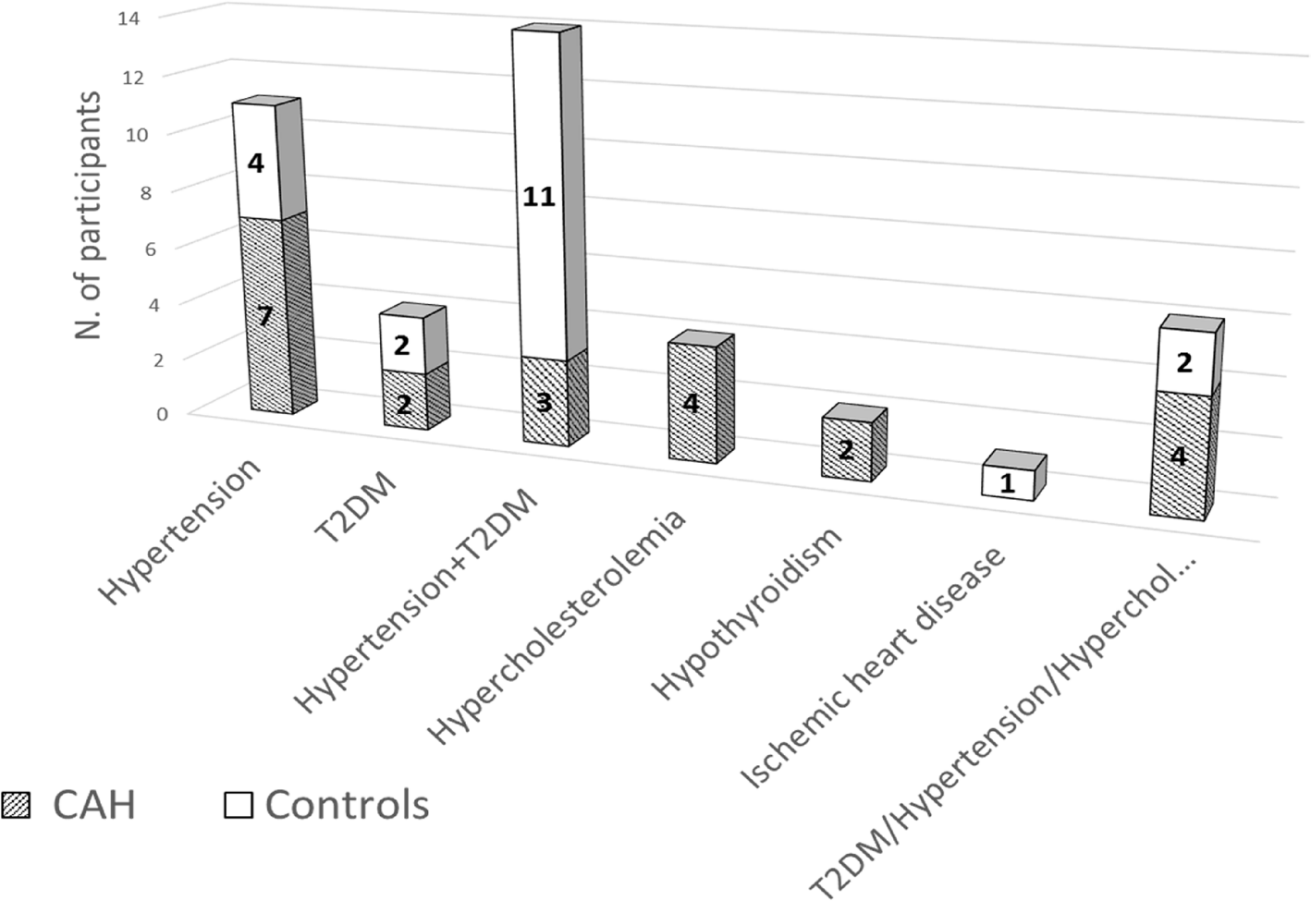
Family history in patients with congenital adrenal hyperplasia and matched controls. T2DM, Type 2 diabetes mellitus

In total, 44 subjects were included (22 patients with 21OHD and 22 matched controls, respectively), 36 (82%) were females, with a mean age of 17.1 ± 5.5 years (range 10–30, 33 (75%) between 10 and 20 years) (Table 1). Of the total, 30 (68%) had already completed puberty (Tanner 5). No patient was excluded due to any of the exclusion criteria. No smokers were found in neither patients nor controls. Glucocorticoid supplementation was present in 17 patients (77%). Of the 9 patients with non-classic CAH, 4 of them received glucocorticoid treatment because they had early pubarche with acceleration of bone age and rapidly progressive puberty. All those without glucocorticoids had the non-classic phenotype. The type of steroid treatment that dominated in the patients was hydrocortisone alone or combined with fludrocortisone, which represented 12 patients (71%), followed by cortisone acetate for 5 patients (29%). The blood pressure was similar between patients with CAH and matched controls (Table 1). Four patients with CAH and four controls had blood pressure levels in the range of prehypertension or hypertension. Family history revealed a history of diabetes mellitus, hypertension, and hypercholesterolemia in more than 50% of the subjects in both groups (Fig. 1).

**Table 1:** Clinical characteristics of 22 patients with congenital adrenal hyperplasia due to 21-hydroxylase deficiency and age-, sex-, body mass index- and Tanner staged matched controls

Variables	Study Groups		*p*-value
	**Patients with CAH** ***n* = 22**	**Matched controls** ***n* = 22**	
**Age (years)**	17.2 ± 5.7	16.9 ± 5.4	0.89
**Sex (n**)	18 (82%) F 4 (18%) M	18 (82%) F 4 (18%) M	1.00
**Weight (kg)**	60.3 ± 16.5	62.7 ± 22.8	0.70
**Height (cm)**	157.5 ± 9.8	159.9 ± 10.5	0.44
**Waist circumference (cm)**	73.5 ± 11	76.1 ± 14.4	0.52
**BMI (kg/m**^**2**^**)**	24.2 ± 6.3	24.1 ± 7.3	0.97
**SBP (mmHg)**	102.5 ± 14.3	107 ± 15	0.31
**DBP (mmHg)**	65 ± 9.6	68 ± 9.6	0.30

*F* Female, *M* Male, *BMI* Body mass index, *SBP* systolic blood pressure, *DBP* diastolic blood pressure

Patients with 21OHD had lower blood glucose levels ​​(*p* < 0.01) but higher TG and the atherogenic index compared to matched controls (both *p* < 0.01). A negative correlation was observed between the atherogenic index and the FMD (*p* = 0.02); as well as with the CIMT (*p* = 0.03). No relationship was found with epicardial fat (*p* = 0.33). All other biochemical tests were similar between patients and matched controls (Table 2).

**Table 2:** Cardiometabolic risk factors in adolescents and young patients with congenital adrenal hyperplasia due to 21-hydroxylase deficiency and controls matched for age, sex, BMI and pubertal stage

**Variables**		**Patients with CAH** *n* = 22	**Controls** *n* = 22	**p**
*Physical examination*				
Excess weight, n (%)		11 (50%)	10 (45%)	1.00
Abdominal Adiposity, n (%)		11 (50%)	9 (41%)	0.76
Blood pressure, n (%)	Normal	18 (82%)	18 (82%)	0.55
	Prehypertensive	2 (9%)	1 (5%)	
	Hypertensive	2 (9%)	3(14)	
*Biochemical tests*				
Blood glucose (mmol/L) mean ± SD		3.9 ± 0.68	4.7 ± 0.64	** < 0.01**
Insulin (µmUI/ml) mean ± SD		15.7 ± 8.8	14.1 ± 3.9	0.45
HOMA-IR mean ± SD		2.8 ± 1.7	2.9 ± 1.0	0.70
Insulin resistance, n (%)		9 (41%)	11 (58%)	0.28
Total cholesterol (mmol/L) mean ± SD		4.0 ± 1.1	3.7 ± 0.7	0.35
High total cholesterol**,** n (%)		2 (9%)	1 (5%)	0.55
LDL (mmol/L) mean ± SD		2.4 ± 1.1	2.2 ± 0.5	0.33
	Elevated	2 (9%)	0 (0%)	0.49
HDL (mmol/L) mean ± SD		1.1 ± 0.3	1.2 ± 0.4	0.15
	Low	10 (46%)	6 (27%)	0.35
Atherogenic index		3.78 ± 1.0	3.0 ± 0.7	** < 0.01**
Triglycerides (mmol/L) mean ± SD		1.1 ± 0.49	0.76 ± 0.29	** < 0.01**
	Elevated	3 (14%)	1 (5%)	0.61

*CAH* congenital adrenal hyperplasia

The epicardial fat mean in adolescents and young patients with CAH was 1.13 ± 1.63 and in matched controls 1.02 ± 0.66 (*p* = 0.48). The presence of epicardial fat had a positive relationship with total cholesterol levels ​​(*r* = 0.679, *p* < 0.01), the time since CAH diagnosis (*r* = 0.462, *p* = 0.03) and the glucocorticoid dose (*r* = 0.499, *p* = 0.04).

Adolescents and young adults with CAH had a discreet tendency of increased mean CIMT (*p* = 0.07) and greater frequency of increased CIMT (*p* = 0.09), i.e., subclinical atherosclerosis and a non-significant higher percentage of endothelial dysfunction (*p* = 0.41) (Table 3). Unfortunately, one patient did not have the CIMT nor FMD done. In the multivariate analysis, the CIMT was directly related to the 17OHP levels (*r* = 0.510, *p* = 0.018), DBP (*r* = 0.444, *p* = 0.04) and the HOMA index (*r* = 0.507, *p* = 0.01), but not with current hydrocortisone equivalent dose (*r* = -0.186, *p* = 0.491). Of the 21 patients with CAH in whom the CIMT were explored, 11 (52%) had it increased. Comparisons between those with CAH and increased vs normal CIMT were made with the only difference being more females in the increased CIMT group (11/11 vs 6/10, *p* = 0.035) (other data not shown).

**Table 3:** Measurements of subclinical atherosclerosis using flow-mediated dilation of the brachial artery and the carotid intima media thickness in adolescents and young adults with congenital adrenal hyperplasia due to 21-hydroxylase deficiency and age-, sex-, body mass index- and Tanner staged matched controls

	Patients with CAH *n* = 21	Matched controls *n* = 22	*p*-value
CIMT (mm)	0.45 (0.4–0.6)	0.42 (0.2–0.5)	0.07
Increased (n)(> 90 p)	11 (52%)	6 (27%)	0.09
CIMT Right (mm)	0.5 (0.3–0.7)	0.4 (0.3–0.6)	0.10
CIMT Left (mm)	0.5 (0.3–0.6)	0.5 (0.3–0.6)	0.98
FMD, media ± SD	10.6 ± 5.7	10.3 ± 6.2	0.83
Endothelial dysfunction (n)	4 (19%)	2 (9%)	0. 41
Epicardial fat, median (range)	1 (5.8)	1 (2.1)	0.64
Increased (n) ≥ 5 mm	2 (9%)	0 (0%)	0.23

*CIMT* Carotid intima media thickness, *p* percentile, *FMD* flow-mediated dilation

In relation to clinical variables and cardiometabolic risk factors, it was found that only triglyceride concentrations had a statistically significant positive association with the time since the CAH diagnosis (*r* = 0.45, *p* = 0.03).

No statistically significant differences were found between those patients with CAH and preserved endothelial function and those with dysfunction (data not shown).

The characteristics of the patients with CAH are shown according to phenotype in Table 4. The patients with the non-classic phenotype were diagnosed later, had shorter time since diagnosis, lower 17OHP levels and higher CIMT values in addition to a tendency to lower hydrocortisone equivalent dose compared to the classic CAH phenotypes (salt-wasting and simple virilizing, respectively).

**Table 4:** Variables related to 21-hydroxylase deficiency according to phenotype

	**Phenotype**			***p*- value**
	**SW** ***n* = 8**	**SV** ***n* = 5**	**NC** ***n* = 9**	
**Age at diagnosis** **(years)**	0.01 (0.01–1)	1 (0.01–8)	12 (6–18)	** < 0.01**
**Time since diagnosis (years)**	14 (10–31)	11 (10–25)	8 (0.8–12)	** < 0.01**
**Hydrocortisone equivalent dose** **mg/d**	37.5 (15–40)	27.5 (15–50)	15 (15–17.5)	0.10
**Hydrocortisone equivalent dose (mg/m**^**2**^**/d)**	20 (12.9–27.1)	17.8 (7.5–31.4)	8.7 (8.7–10.6)	0.07
**Testosterone (nmol/L)**	2.9 (0.5–6.4) F 4.0 M	4.2 (1.1–9.2) F 7.0 (0.8–13.1) M	2.9 (0.5–6.4) F 4.0 M	0.91
**17OHP (ng/mL)**	12.4 (3.1–14)	13 (1.5–13.2)	2.4 (0.3–13)	**0.03**
**CIMT (mm)**	0.46 (0.4–0.55)	0.41(0.3–0.5)	0.52 (0.45–0.6)^a^	**0.05**
**FMD** **(%)**	9.65 (0.0–17.96)	11.55 (4.29–19.47)	10.91 (2.61–19.85)^a^	0.84
**Epicardial fat** **(mm)**	1.44 (0.0–5.8)	0.62 (0.0–1.1)	1.14 (0.0–5.0)	0.82

*SW* Salt-wasting, *SV* simple virilizing, *NC* non-classic form, *170HP* 17-hydroxyprogesterone, *CIMT* Carotid intima media thickness, *FMD* flow-mediated dilation

^a^*n* = 8. *F* female, *M* male

## Discussion

This study investigated subclinical atherosclerosis in Cuban patients for the first time, measured as CIMT, had a tendency to be more common in CAH compared to controls matched by age, sex, BMI and Tanner stage. CIMT was associated with hormonal control, diastolic blood pressure and insulin resistance in patients with CAH. However, even though endothelial dysfunction was more than twice as common in these young patients with CAH than in matched controls, this was not statistically significant, probably due to the limited number of participants and the young age. Furthermore, comparing patients with CAH with controls matched by BMI is quite unique in the field of cardiovascular risk in CAH and probably underestimate the differences between patients with CAH and general population.

In the management of CAH it is necessary to evaluate the short- and long-term outcomes, especially cardiovascular and metabolic complications which have been shown to be increased in some studies [6, 10, 26]. This topic has gained in importance in recent years, although not much data have been collected in pediatric patients [27].

The main objective of therapy in patients with CAH is to replace the deficient hormones and normalize the adrenal androgen levels, for which glucocorticoids and mineralocorticoids are used [1, 2]. It is a challenge to maintain balance between hyperandrogenism and hypercortisolism. On the one hand, suboptimal doses increase adrenal androgen production, accelerate skeletal maturation, produce a loss of growth potential and increase the risk for adrenal crisis. On the other hand, in over-treated patients, growth is suppressed, blood pressure is increased, iatrogenic Cushing and early cardiovascular complications may appear [13].

In relation to the biochemical parameters, the lower fasting glucose concentrations in patients with CAH compared to controls is probably a sign of cortisol insufficiency in the morning and have also been reported in other studies [9, 13]. Moreover, the epinephrine deficiency demonstrated in CAH may also have contributed [13].

Given the increase in cardiovascular risk in these patients, there is currently a growing interest in detecting vascular disease in its subclinical stage, since intervening in this period is crucial to prevent future cardiovascular events. Among the most used methods for its detection are FMD and CIMT [8, 22, 23]. The study of endothelial function has gained great relevance in medical practice, because dysfunction is an early precursor in the pathophysiology of atherosclerosis [7–13].

Endothelial function can be described as a barometer of cardiovascular health, useful for directing patient management in a personalized way and evaluating therapeutic strategies [28]. Although no significant differences were found in the frequency of endothelial dysfunction in the present study, probably due to limited power and the young age of the participants, endothelial dysfunction was present in twice as many patients compared to matched controls. It should be noted that we matched controls concerning BMI and this probably underestimated the differences between patients with CAH and the general population.

Increased CIMT, is also considered a marker of subclinical atherosclerosis [29], and has recently become a highly valuable test due to multiple clinical trials that have demonstrated its relationship with the early detection of cardiovascular disease [25]. This is why the American Heart Association recommends it as a non-invasive imaging study to detect atherosclerosis [30]. In our study, the CIMT was only non-significantly higher in patients with CAH, where more than half showed increased thickness (> 90th percentile), which is an indication of a subclinical atherogenic process [31]. The associations between CIMT and 17OHP levels, diastolic blood pressure and the HOMA index were striking and have been shown by others as well [27]. However, they did not, similar to us, find a statistical difference in CIMT between their children with CAH compared to healthy controls [27]. The differences that have been found between patients with CAH and controls have been associated with disease control, such as duration of treatment and androgen levels, which act on the function of the arterial wall [32]. Nevertheless, when we compared different clinical data, such as age at diagnosis of CAH, duration and current dose of glucocorticoid supplementation and clinical signs of hyperandrogenism in our patients with increased with those patients with normal CIMT we could not find any significant findings. In contrast, Kim et al. did find significant associations with these parameters [33]. However, we did find a positive relationship between CIMT and 17OHP. Thus, the poorer hormonal control, the more subclinical atherosclerosis. Moreover, our adolescents and young adults with the non-classic phenotype had increased CIMT compared to the other more severe phenotypes which could indicated that prolonged untreated hyperandrogenism plays a role in the development of atherosclerosis. However, this result should be taken with reserve considering the small size of the groups.

In the present study, 18% of patients with CAH had prehypertension or hypertension which was similar to matched controls. Blood pressure measurements in patients with CAH have varied [34], even though a meta-analysis indicated increased blood pressure in patients with CAH compared to controls. The blood pressure in CAH may depend on the age, glucocorticoid and mineralocorticoid dose, hormonal control, delay of CAH diagnosis, other risk factors such as obesity and so on [13, 35].

Epicardial fat has emerged in recent years as a relevant target, towards which numerous interventions and therapies are directed today. There are few reports evaluating epicardial fat in patients with CAH. Kim et al. [36], have been pioneers and have found, similar to the present study, a high epicardial fat, and an association with duration of CAH, waist circumference and the dose of glucocorticoids used [29].

### Limitations

Like all studies, also the current study had limitations. The limited number of included patients with CAH, but still a decent size compared to previous studies [12, 26], the wide age range, only few males, not having access to the total glucocorticoid and androgen exposure during the entire life as well as the economic constraints preventing us from analyzing more biochemical tests such as androstenedione and DHEAS were some of them. The limited number of included participants made it difficult to reach statistical significance in the differences found. However, in contrast to most previous studies investigating cardiovascular risk factors in CAH [10, 26], we matched controls for BMI. This probably underestimated the differences since obesity is more common in CAH than in the general population in most previous studies [8, 10, 29, 30]. It should be noted that obesity is a risk factor for subclinical atherosclerosis and in spite of us controlling for BMI we did find tendencies of subclinical atherosclerosis in adolescents and young adults with CAH. Moreover, atherosclerosis develop over time and an older study population would more likely find larger differences. The results may in some respects be generalized to other variants of CAH, however, variants with known increased mineralocorticoid levels and thus hypertension such as 11β-hydroxylase deficiency may have even higher cardiovascular risk [37]. Another strength, apart from the matched controls, is that the study is prospective while retrospective studies are the most common in the field of CAH. Furthermore, this study was performed in a non-high-income country with economical constraints, while most previous studies concerning cardiovascular risk in CAH have been done in high-income countries [29]. Moreover, the current population is characterized by a mixture of fundamentally European and African genes which has not been studied so much previously. This gives some new perspectives to CAH care and management.

## Conclusions

Adolescents and young adults with CAH had a strong tendency of increased CIMT compared to age-, sex-, Tanner stage and BMI matched controls. CIMT was directly related to 17OHP, diastolic blood pressure and HOMA-index. Epicardial fat was positively associated with total cholesterol levels, duration of CAH and the glucocorticoid dose. Non-classic phenotype may be more affected. Adolescents and young adults with CAH should be regularly assessed for cardiovascular risk and non-invasive methods such as measurement of epicardial fat, CIMT and FMD.

## Data Availability

The original contributions presented in the study are included in the article. Further inquiries can be directed to the corresponding author.
